# The association between major dietary patterns at dinner and obesity in adults living in Tehran: A population-based study

**DOI:** 10.34172/jcvtr.2020.45

**Published:** 2020-11-28

**Authors:** Zahra Akbarzade, Kurosh Djafarian, Cain C. T. Clark, Azadeh Lesani, Hossein Shahinfar, Sakineh Shab-Bidar

**Affiliations:** ^1^Department of Community Nutrition, School of Nutritional Sciences and Dietetics, Tehran University of Medical Sciences (TUMS), Tehran, Iran; ^2^Department of Clinical Nutrition, School of Nutritional Sciences and Dietetics, Tehran University of Medical Sciences, Tehran, Iran; ^3^Centre for Sport, Exercise, and Life Sciences, Coventry University, Coventry, CV15FB, UK

**Keywords:** Dinner Pattern, Obesity, Adults

## Abstract

***Introduction:*** Findings of studies on the association between evening meal and obesity are inconclusive. Thus, we sought to investigate the association between major dietary patterns at evening meal and obesity among apparently healthy adults in Tehran.

***Methods:*** This cross-sectional research was conducted using 833 adult men and women who lived in Tehran (age range: 20-59 years). Their dietary intake was evaluated by three, 24-h dietary recalls(24hDRs), and major patterns were identified using exploratory factor analysis. The association between major dietary patterns at dinner with general and central obesity was assessed using logistic regression analysis.

***Results:*** We identified 3 major dietary patterns at dinner including "prudent", "potatoes and eggs" and"Western" patterns. There was no significant relationship between prudent and general obesity (OR:0.76, 95% CI = 0.21, 1.15, P value = 0.20), and, a significant association was not observed between potatoes and eggs and general obesity (OR: 0.89, 95% CI = 0.60, 1.32, P value = 0.57) also, there was no significant relationship between Western dietary pattern and general obesity in this study (OR: 0.95,95% CI = 0.63, 1.43, P value = 0.82). Further analyses showed that there was no significant relationship between central obesity with any of the dietary patterns.

***Conclusion:*** The results of this study do not support a possible relationship between major dietary patterns at dinner with general and central obesity. However, the presented findings should be confirmed in prospective studies.

## Introduction

Incidence of obesity has more than doubled, globally, since 1980.^1^ In Iran, particularly in the northern region of the country, over half of the adult population are reportedly overweight or obesity.^2^ Incidence of obesity in not uniform across different regions of Iran, and is strongly affected by socioeconomic status and demographic factors such as age, sex, education, occupation, and marital status.^3^ Obesity negatively affects almost all physiological functions of body and is a major risk factor related to innumerable non–communicable diseases (NCD).^4^ Moreover, obesity and overweight are associated with excess morbidity and early mortality.^5^

Preventing obesity is one of the most effective approaches to control the increasing rate of cardiovascular disease (CVD), stroke, hypertension, and cancer, globally .^4^ Furthermore, diet and physical activity are considered the most important determinants of individuals’ lifestyle, and have an acknowledged impact on obesity, metabolic, diabetes and cardiovascular risk.^6,7^ The majority of published papers on the topic investigated the association of diet and obesity, with a particular emphasis on foods or nutrients. In recent years, using a posteriori dietary patterns to find associations between diet and chronic disease has been advocated. Dietary pattern is considered a preferable method for defining dietary intake and comprehending eating behavior^8^; indeed, it has been indicated that a food pattern high in fruits, vegetables and fiber, and low in meat and fat was inversely associated with risk of obesity.^9^ In contrast, a Western dietary pattern (high consumption of red meat and processed food) was also related to higher chance of being overweight or obese .^10^ In Iran, several dietary patterns have been purported to be associated with general or central obesity.^11,12^ Previous dietary pattern analyses have generally focused on habitual diet; however, there are currently few studies that have investigated specific meal patterns.^13^ Indeed, understanding how food selections are made at meal times can be a critical start-point in developing dietary strategies for better food choices. Dinner is typically the most energy-dense meal of the day^14^; however, previous research has shown that a reduction in daytime energy intake may be compensated by increases in evening and mid-afternoon intake (dinner-time)^15^, which, in turn, is associated with skipping, or reduced consumption of, breakfast, and can hinder diet induced thermogenesis (DIT)^16^ and insulin sensitivity.^17^

A number of studies have shown a direct relationship between eliminating the dinner meal and obesity.^18^ On the other hand, other investigations have reported the opposite, so that people who have dinner are at higher risk of obesity.^19^ Some studies have also shown that those who eat dinner are less likely to develop obesity, overweight, abdominal obesity and abnormal HDL levels. Also, people who skipped dinner were at higher risk for obesity, overweight, truncal obesity and abnormal HDL levels. In addition, eating dinner showed a significant negative relationship with a number of risk factors for cardiovascular disease.^20^ Moreover, in recent years in Iran we faced nutrition transition and changing ordinary daily eating habits. In other words, modernizations lead to a dissolution of family meals, increased snacking and consumption of fast food and finally considering dinner meal as main meal to get together as family. Furthermore, the association of major dietary pattern at dinner, as a main meal, and obesity has not previously been investigated in Iran. Thus, the present study sought to investigate the relationship between major dietary patterns at evening meal and obesity among apparently healthy adults in Tehran.

## Material and methods

### Participants

The present study was a cross-sectional study on people aged 20 to 59 years and was conducted in Tehran from 2018 to 2019. The following formula was used for sample size calculation: n = (pqz2)/E2. Considering the total prevalence 65% for overweight and obesity^21^, an error coefficient of d=0.04 and at a level of 0.05, the sample size of 546 people was obtained. With design effect of 1.5 and in order to compensate for the potential exclusion of participants due to under- and over-reporting of total energy intake, or attrition due to other reasons, the final sample size of 850 participants was selected for inclusion. Two-stage cluster sampling was used to recruit people from health centers. At first, we classified the health centers into 5 different districts containing North, South, East, West and Central. In the next step, a list of all health centers which exist in each zone was provided. Then we randomly chose forty health centers (considering budget and time limitation). Then, to obtain the number of subjects in each health center we divided the total number of sample size (=850) into the number of health centers (=40). The study inclusion criteria were; age range of 20-59, willingness to take part in the study, self-certified to be healthy, and being a resident of Tehran. The sample collection was facilitated by the coordination of the Health Bureau of the Municipality of Tehran and the cooperation of the health centers of Tehran. All necessary explanations about project were given to the participants. All procedures were conducted in accordance with the ethical standards of the Tehran University of Medical Sciences (Ethic Number: IR.TUMS.VCR.REC.1397.157), who approved the protocol and informed consent form. All participants signed a written informed consent prior to the start of the study.

### Dietary assessment

We used a 24-h dietary recall to evaluate the dietary intake of subjects. The first recall was performed during the participants’ first visit at the health center. The other two 24-h dietary recalls were conducted at random days, including weekends, by telephone. All 24-h dietary recalls were carried out by trained dietitians. Any food or beverage that the participant had consumed during the denominated meal was considered. For our analysis, daily intake of all food items from 24-h dietary recall was computed and then consumed foods were converted into grams using household measures.^22^ Dinner was defined as a large meal eaten between 17:00 and 23:00.^23^ Food items were grouped to the fourteen groups according to their nutritional values, Iranian consumption habits, literature review and experience of the research team in previous studies. Some individual food items that consisted of separate items (e.g. eggs) or that represented especial dietary habits (such as, potatoes) were kept as a single food group.

### Anthropometric measurements and blood pressure

Weight was measured using digital scale for adults (Seca model 808, measurement accuracy +/-100g).^24^ Participant’s height was measured, unshod, using a wall stadiometer with a sensitivity of 1mm (Seca, Germany).^24^ Body Mass Index (BMI) was calculated by dividing weight in kilograms by the square of height in meters. General obesity was defined as BMI ≥30 kg/m2.^25^ Waist circumference (WC) was measured with a tape measure to the nearest 0.1 cm between the iliac crest and the lowest rib during exhalation, and hip circumference was recorded at maximal point, over light clothing, using a non-stretch tape measure, without exerting any pressure on body surface. Central obesity was defined as WC≥102 in men and WC≥88 in women, as well as a waist-hip ratio >0.90 for males and >0.85 for females.^26^

Systolic and diastolic blood pressure was measured by a trained physician, in the right arm, with a standard mercury sphygmomanometer, after the participant had been sitting quietly for 15 min. This measurement was followed by a second measurement 1-2 min later, and the mean of the two was calculated.

### Assessment of other variables

We used the International Physical Activity Questionnaire (IPAQ) to obtain data on physical activity.^27^ Using standard guidelines these data were expressed as metabolic equivalent hours per week (MET-h/week).^28^ Subjects completed a questionnaire designed to assess the participants’ demographics, such as age (year), BMI (kg/m^2^), education level (illiterate, under diploma, diploma, educated), marital status (married or other), occupation (employed or unemployed), medical condition (healthy or underlying disease), smoking status (never smoked, former smoker, current smoker), Life-style (living alone, with someone).

### Identification of dietary patterns

To identify dietary patterns, we used exploratory factor analysis (EFA). To perform the exploratory factor analysis, we first measured the appropriateness of the data using the Bartlett’s test of sphericity test and the Kaiser-Meyer-Olkin criterion (more than 0.6). Dinner-derived dietary patterns were extracted using factor analysis and using a standard principal component analysis method. We used the estimated intake of fourteen food groups (g/d) as input variables in factor analyses. Factors were rotated with an orthogonal (varimax) rotation to improve interpretability and minimize the correlation between the factors. The number of factors retained from each dietary pattern classification method was determined by eigenvalues (>1.10), scree plots, and factor interpretability. Higher loadings (≥ 0.2) show that the food shares more variance with that factor. Labelling of the factors was primarily descriptive and based on our interpretation of the data and past studies that found similar dietary meal patterns .^29^ Then, scores for dinner patterns were calculated as the sum of the products of the factor loading coefficients and summing intakes of food groups weighted by their factor loadings.^30^ Each participant received a factor score for each identified pattern.

### Statistical analysis

Kolmogorov–Smirnov test was performed for normality of the distributions of variables. Of the initial 850 participants, 17 were excluded due to missing data. Therefore, 833 individuals were used in the final analysis. Participants were categorized based on tertiles of major dietary pattern scores. Homogeneity of variance assumption was examined with Levene’s F tests. One-way analysis of variance (ANOVA) and chi-square tests were used to compare general characteristics of participants across tertiles of dietary patterns. To test collinearity statistics, we used the regression models and found that the variance inflation factor for independent variable was less than 10. Multiple logistic regression analysis was used to determine the association of dinner-derived dietary patterns with obesity in crude and adjusted models. Confounders were selected based on literature review including age (years), gender (male or female), physical activity level, smoking status (never smoke or former/current smoker), and total energy intake. All statistical analyses were performed using the IBM SPSS Statistics (version 19, IBM Company, Armonk, NY, USA). We considered *P*< 0.05 to represent the threshold for statistical significance.

## Results

Of 833 participants, 165 were men (19.6 %) and 670 were women (80.4%) with an average age of 42.30±10.98 years. 80.7% were married and 90.4% lived with others.35.2% had a university degree, 52.3% had a homeowner and 80.6% had an income below 2 million. In addition, the present study showed that 25.3% and 44.8% of subjects had general and central obesity, respectivelyFigure 1. Using factor analysis, three major dietary patterns at dinner meal were identified, and presented in Table 1 and Figure 2. The first dietary pattern was defined as “Prudent”, which was rich in bread and grains, fats, poultry and fish, and salt, as well as low consumption of fruits and vegetables, nuts and legumes; the second dietary pattern, “egg and potato”, that included a high consumption of potato and egg but low dairy and nuts and legumes consumption, and the third dietary pattern, “Western”, which was rich in red or processed meat, sauces, soft drinks and bread and grains, and lower consumption of poultry and fish. These three dietary patterns defined 38.746% of the total variance. Correlation analysis showed that there were strong positive correlations between fat and salt (*P* ≤ 0.001) and between potato and egg (*P* ≤ 0.001), and, in contrast, we found strong negative correlations between nuts and legumes and eggs (*P* ≤ 0.001), and between eggs and fish and poultry (*P* ≤ 0.001). There were also correlations between red meat and processed meat and poultry and fish (*P* ≤ 0.001) (Figure 3).

**Figure 1:**
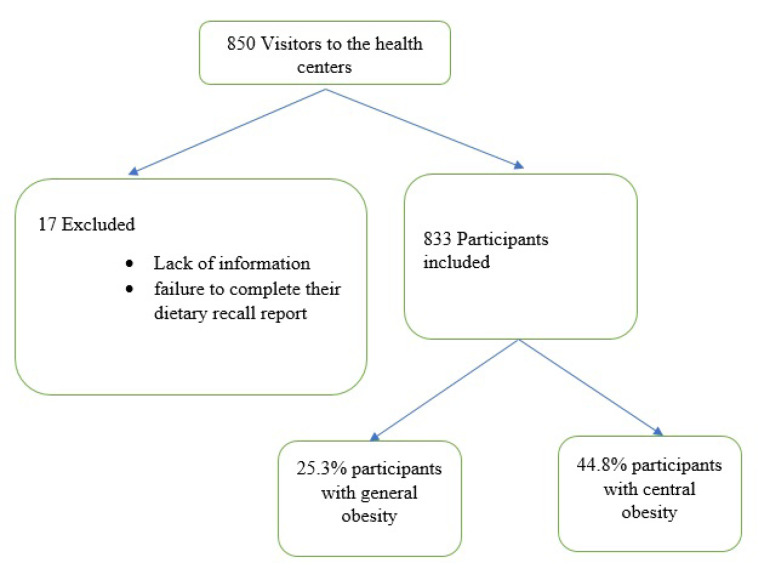
A flow chart showing inclusion and exclusion criteria for participants study

**Table 1:** Food groups used in the factor analysis and factor loadings for each of the identified dietary patterns ^a^

**Food groups**	**Food items**	**Dietary pattern**		
		**Prudent**	**Potatoes and eggs**	**Western**
Bread and grains	White breads (lavash, baguettes), noodles, pasta, rice, toasted bread, white flour, Dark breads (e.g., barbari, sangak, taftun)	0.606	-	0.357
Salt	Salt	0.756	-	-
Dairy products	Low-fat milk, skim milk, low-fat yogurt, cheese, Kashk, yogurt drink, High-fat milk, high-fat yogurt, cream cheese, cream, dairy fat, ice cream, others	-	-0.314	-
Egg	Eggs	-	0.800	-
Fat	Hydrogenated fats, animal fats, butter, oils Olive oil, vegetable oils, olives	0.691	0.263	-
Potato	Potatoes	-	0.727	-
Red meat and processed meats	Sausage, hamburger, other	-	-	0.764
Organ meats	Heart, kidney, liver, tongue, brain,	-	-	0.204
Fruits and vegetables	Melon, watermelon, honeydew melon, plums, prunes, apples, cherries, sour cherries, peaches, nectarine, pear, fig, date, grapes, kiwi, pomegranate, strawberry, banana, persimmon, berry, pineapple, oranges, dried fruits, all juices, Cauliflower, carrot, tomato and its products, spinach, lettuce, cucumber, eggplant, onion, greens ,green bean, green pea, squash, mushroom, pepper, corn, garlic, turnip, others	0.217	-	-
Legumes and nuts	Peanuts, almonds, pistachios, hazelnuts, roasted seeds, walnuts, Lentils, split pea, beans, chick pea, fava bean, soy, others	0.332	-0.350	-
Soft drink	Soft drinks			0.593
Pickle		-	0.374	0.372
Sauces	Mayonnaise, Ketchup, tomato paste			0.525
Poultry and fish	Chicken and fish	0.396	-	-0.269

^a^Factor loadings of<0.2 have been removed to simplify the table

**Figure 2:**
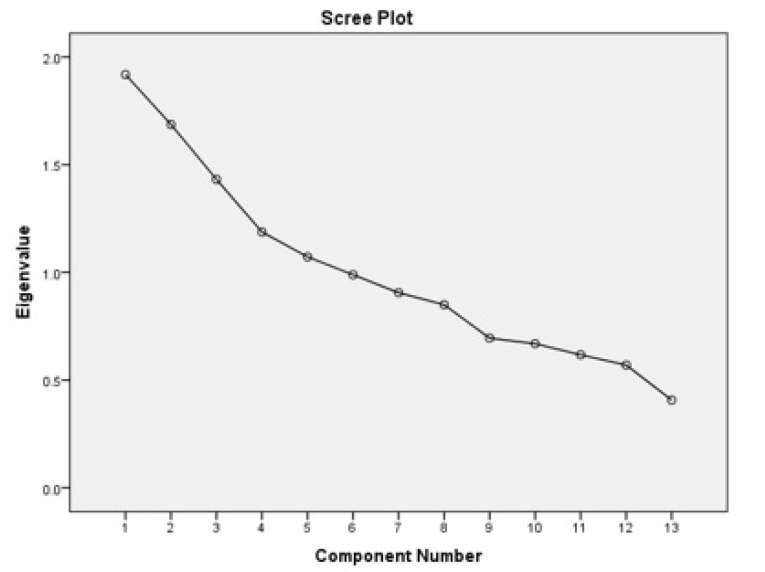
Scree plot for identification of dietary patterns by principal component analysis

**Figure 3:**
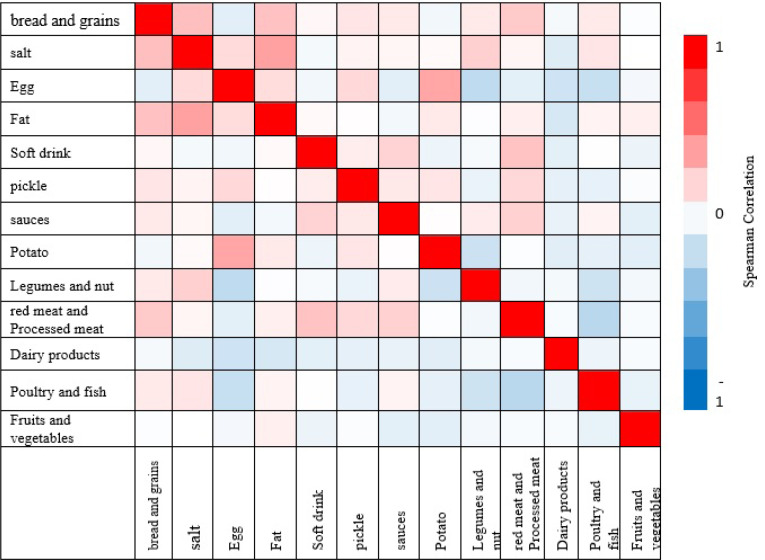
Heat map showing the Spearman correlation matrix for dinner food intake in grams (n = 833) by food group. The color corresponds to the strength of correlations (red: positive correlation; white: no correlation; blue: nega

Table 2 shows the distribution of the qualitative and quantitative variables among the tertiles of dietary pattern. The results showed that distribution of qualitative characteristics were not significant across tertiles of a “prudent” dietary pattern, and “potatoes and eggs”. However, the distribution of smoking status was different among the tertiles of the “Western” dietary pattern (*P* = 0.04). Adherence to the “prudent” dietary pattern was associated with a significant decrease in mean intake of dairy products (*P* ≤ 0.001) and a significant increase in mean intake of fruit (*P* ≤ 0.001), vegetables (*P* ≤ 0.001), and grains (*P* ≤ 0.001). Additionally, mean intake of dairy products decreased across tertiles of the egg and potato dietary pattern (*P* ≤ 0.001). In addition, the intake of the grains and meat was significantly decreased across tertiles of the “Western” dietary pattern (*P* ≤ 0.001), and the intake of fruit significantly increased (*P* ≤ 0.001). The mean of energy intake in the third tertile of all three dietary patterns was higher compared to the first tertile (*P* < 0.001).

**Table 2:** Characteristics of the study participants by tertiles (T) of dinner pattern scores

**Tertiles of dietary patterns**												
	**Prudent**				**Potatoes and eggs**				**Western**			
	**T1**	**T2**	**T3**	***P*** ** value**	**T1**	**T2**	**T3**	***P*** ** value**	**T1**	**T2**	**T3**	***P*** ** value**
**Participants**	**266**	**283**	**284**		**266**	**283**	**284**		**266**	**283**	**284**	
Sex, n (%)				0.82				0.22				0.36
Male	49(30.1)	56(34.4)	58(35.6)		61(37.4)	44(27)	58(35.6)		46(28.2)	60(36.8)	57(35)	
Female	217(32.4)	228(34)	225(33.6)		219(32.7)	227(33.9)	224(33.4)		226(33.7)	218(32.5)	226(33.7)	
Activity score				0.42				0.25				0.24
Low	147(33.6)	147(33.6)	144(32.9)		149(34)	145(33.1)	144(32.9)		139(31.7)	145(33.1)	154(35.2)	
Moderate	94(29.7)	112(35.3)	111(35)		96(30.3)	107(33.8)	114(34)		102(32.2)	114(36)	101(31.9)	
High	25(33.3)	22(29.3)	28(37.3)		34(45.3)	18(24)	23(30.7)		29(38.7)	19(25.3)	27(36)	
Education, n (%)				0.65				0.33				0.81
Educated	92(31.4)	108(36.9)	93(33.7)		91(31.1)	98(34.4)	104(35.5)		104(35.5)	92(31.4)	97(33.1)	
Occupation, n (%)				0.81				0.81				0.22
Employee	101(32.9)	106(34.5)	100(32.6)		107(34.9)	100(32.6)	100(32.6)		90(29.3)	103(33.6)	114(37.1)	
Marriage, n (%)				0.12				0.15				0.35
Married	212(31.5)	221(32.9)	239(35.6)		234(34.8)	209(31.1)	229(34.1)		215(32)	232(34.5)	225(33.5)	
Life-style, n (%)				0.47				0.60				0.59
Living alone	18(39.1)	16(34.8)	12(26.1)		15(32.6)	17(37)	14(30.4)		17(37)	13(28.3)	16(34.8)	
With someone	248(31.6)	267(34)	270(34.4)		264(33.6)	253(33.2)	268(34.1)		255(32.5)	263(33.5)	267(34)	
Smoking, n (%)				0.96				0.36				0.04
Current smoker	8(28.6)	9(32.1)	11(39.3)		12(42.9)	10(35.7)	6(21.4)		6(21.4)	13(46.4)	9(32.1)	
*Medical condition, n (%)				0.51				0.68				0.49
Under lying disease	118(33.4)	123(34.8)	112(31.7)		115(32.6)	121(34.3)	117(33.1)		120(34)	121(34.3)	112(31.7)	
Obesity n (%)	70(32.7)	82(38.3)	62(29)	0.15	73(34.1)	72(33.6)	69(32.2)	0.81	70(32.7)	72(33.6)	72(33.6)	0.98
**Mean ±SD**												
Age(years)	42.65±11.08	42.16±11.1	42.11±10.7	0.55	42.35±10.7	42.59±11.2	44.9±11.02	0.69	42.8±11.7	42.07±10.7	42.07±10.4	0.44
Weight (kg)												
Systolic blood pressure(mmHg)	116.10±21.7	116.3±21.13	117.11±19.2	0.55	116.9±18.7	114.11±21.9	118.5±20.17	0.33	116.9±19.5	116.26±20.8	116.4±20.8	0.77
Diastolic blood pressure(mmHg)	78.76±13.5	87.27±13.65	78.86±12.9	0.32	78.01±13.05	77.39±15.30	79.49±10.9	0.18	78.19±13.99	77.81±12.5	78.9±13.6	0.52
Dietary and nutrient intakes												
Fruit	41.36±44.61	51.42±50.14	57.35±54.33	<0.001	52.84±53.55	52.61±50.38	45.32±46.65	0.07	59.20±53.55	48.19±47.72	43.58±48.52	<0.001
Vegetable	71.09±46.64	84.91±47.45	89.91±53.81	<0.001	79.85±49.42	83.82±49.45	82.97±51.24	0.46	85.52±52.71	80.48±49.76	80.69±47.59	0.25
Dairy	47.55±39.70	39.42±31.17	34.21±32.14	<0.001	55.96±37.95	34.58±29.52	30.09±30.69	<0.001	38.61±33.25	40.66±34.40	41.41±36.71	0.34
bread and grains	72.65±38.59	102.6±35.33	131.6±47.72	<0.001	107.6±50.81	101.51±44.81	99.5±45.96	0.04	79.86±39.01	105.4±40.8	122.5±51.13	<0.001
Meats	13.68±20.44	12.69±16.88	12.35±16.58	0.38	12.64±19.05	13.32±18.67	12.72±16.20	0.95	1.69±3.91	9.09±10.67	27.39±21.95	<0.001
Carbohydrate	70.38±27.63	73.27±31.41	73.67±29.95	0.19	73.75±31.24	72.07±30.19	71.61±27.80	0.39	73.78±34.02	71.81±27.24	71.89±27.71	0.45
Protein	19.68±8.10	20.20±7.97	20.53±7.42	0.20	20.36±8.14	20.28±7.96	19.81±7.40	0.40	20.49±8.24	19.22±6.77	20.72±8.32	0.72
Fat	18.15±7.96	19.08±10.37	19.13±8.39	0.20	18.44±10.04	18.42±8.36	19.52±8.34	0.15	18.74±8.70	18.60±8.77	19.06±9.48	0.68
Total energy intake(kcal/day)	464.05±213.03	527.9±187.4	582.9±188.7	<0.001	512.02±194.07	486.08±187.7	577±221.8	<0.001	406.6±206.3	501.08±149.5	613.3±213.9	<0.001

Data are presented as mean ± standard deviation or n (%).P-values obtained using Chi-square test. * Diabetes, Hypertension, Dyslipidemia, Cardiovascular disease, Cancer and Respiratory disease

Table 3 indicates the multivariable-adjusted means for anthropometric measures and indexes across tertiles of dietary pattern scores.The mean of weight was significantly increased across the “Western” dietary pattern, and the significant increase remained significant following adjustment for age (*P* = 0.03), as well as age, marital status, education, physical activity, and smoking (*P*= 0.04).

**Table 3:** Multivariable-adjusted means for anthropometric measures and indexes across tertiles (T) of dinner pattern scores

	**Prudent**				**Potatoes and eggs**				**Western**			
**Characteristics**	**T1**	**T2**	**T3**	***P*** ** value**	**T1**	**T2**	**T3**	***P*** ** value**	**T1**	**T2**	**T3**	***P*** ** value**
	**(n=266)**	**(n=283)**	**(n=284)**		**(n=266)**	**(n=283)**	**(n=284)**		**(n=266)**	**(n=283)**	**(n=284)**	
					**Mean±SD**							
Weight (kg)	71.75±14.13	72.48±14.44	72.14±13.17	0.74	72.55±14.80	71.60±13.17	72.23±13.71	0.78	70.42±12.82	72.94±13.02	72.99±15.55	0.03
Model 1	71.71±0.85	72.50±0.82	72.16±0.82	0.80	72.56±0.83	71.56±0.84	72.26±0.82	0.68	70.37±0.83	72.96±0.83	73.01±0.82	0.03
Model 2	71.62±0.84	72.64±0.82	72.22±0.82	0.68	72.57±0.82	71.49±0.83	72.43±0.82	0.60	70.44±0.83	72.95±0.82	73.07±0.82	0.04
Model 3	72.87±0.94	72.63±0.81	71.05±0.90	0.34	72.31±0.86	71.62±0.84	72.57±0.85	0.71	70.95±0.88	72.99±0.82	72.54±0.86	0.23
BMI (kg/m2)	27.32±5.22	27.68±6.88	27±4.46	0.35	27.40±5.42	27.36±4.74	27.24±4.45	0.93	27.37±7.17	27.38±4.41	27.24±4.89	0.95
Model 1	27.27±0.33	27.43±0.33	27.27±0.33	0.92	27.41±0.33	27.29±0.33	27.26±0.33	0.94	27.27±0.33	27.43±0.33	27.27±0.33	0.92
Model 2	27.19±0.33	27.78±0.32	27.03±0.32	0.23	27.44±0.32	27.27±0.33	27.29±0.32	0.92	27.32±0.33	27.43±0.33	27.27±0.32	0.92
Model 3	27.51±0.37	27.77±0.32	26.73±0.36	0.10	27.40±0.34	27.27±0.33	27.32±0.34	0.96	27.43±0.35	27.43±0.33	27.15±0.34	0.81
Waist-circumference(cm)	89.31±11.87	89.15±13	88.88±11.22	0.91	89.21±12.89	88.30±11.6	89.83±11.5	0.31	88.34±11.15	89.66±12.18	89.34±12.74	0.39
Model 1	89.22±0.71	89.19±0.68	88.94±0.69	0.95	89.21±0.69	88.20±0.70	89.90±0.69	0.22	88.16±0.70	89.75±0.69	89.41±0.69	0.24
Model 2	89.11±0.71	89.43±0.68	88.92±0.68	0.86	89.21±0.69	88.20±0.70	90.02±0.68	0.18	88.29±0.70	89.75±0.69	89.40±0.68	0.31
Model 3	90.15±0.79	89.41±0.68	87.97±0.75	0.17	89.06±0.72	88.19±0.70	90.17±0.71	0.14	88.61±0.74	89.74±0.69	89.10±0.72	0.53
Waist to hip ratio	0.87±0.17	0.86±0.09	0.86±0.07	0.75	0.86±0.07	0.87±0.17	0.85±0.11	0.55	0.87±0.17	0.86±0.08	0.86±0.09	0.61
Model 1	0.87±0.008	0.86±0.008	0.86±0.008	0.72	0.86±0.008	0.87±0.008	0.85±0.008	0.54	0.87±±0.008	0.86±0.008	0.86±0.008	0.59
Model 2	0.87±0.008	0.86±0.008	0.86±0.008	0.79	0.86±0.008	0.87±0.008	0.85±0.008	0.54	0.87±±0.008	0.86±0.008	0.86±0.008	0.53
Model 3	0.87±0.009	0.86±0.008	0.86±0.009	0.68	0.86±0.008	0.87±0.008	0.86±0.008	0.69	0.87±±0.009	0.86±0.008	0.86±0.008	0.67

Model 1: adjusted for age (continuous) Model 2: additionally adjusted for marital status, education, physical activity, smoking Model 3: further adjustment for dietary intake of fruits, vegetables, dairy, grains, energy intake *P* values obtained using ANCOVA test.

Unadjustedand adjusted odds ratios (OR) for the participants’ general obesity across the tertile of dietary patterns are presented in Table 4. According to our findings, there was no significant relationship between prudent and general obesity (OR: 0.76, 95%CI = 0.21, 1.15, *P*value = 0.20), and, a significant association was not observed between potatoes and eggs and general obesity (OR: 0.89, 95%CI = 0.60, 1.32, *P*value = 0.57) also, there was no significant relationship between Western dietary pattern and general obesity in this study (OR: 0.95, 95%CI = 0.63, 1.43, *P* value = 0.82).

**Table 4:** Odds ratio (OR)s and 95% confidence intervals(CI) for general obesity (BMI≥30) across tertiles (T) of dietary patterns score

	**Tertiles of dietary pattern score**							
	**T1**	**T2**		***P*** ** value**	**T3**		***P*** ** value**	**P trend**
		**OR**	**95% CI**		**OR**	**95% CI**		
	**(n=266)**	**(n=283)**			**(n=284)**			
Prudent								
Model 1	1.00 (Ref)	1.21	(0.77,1.62)	0.54	0.77	(0.52,1.13)	0.19	0.20
Model 2	1.00 (Ref)	1.14	(0.78,1.67)	0.47	0.76	(0.51,1.13)	0.18	0.12
Model 3	1.00 (Ref)	1.14	(0.78,1.67)	0.48	0.76	(0.21,1.15)	0.20	0.13
Potatoes and eggs								
Model 1	1.00 (Ref)	1.02	(0.70,1.48)	0.90	0.89	(0.61,1.31)	0.57	0.58
Model 2	1.00 (Ref)	1.02	(0.69,1.49)	0.90	0.89	(0.60,1.31)	0.57	0.76
Model 3	1.00 (Ref)	1.01	(0.69,1.48)	0.93	0.89	(0.60,1.32)	0.57	0.78
Western								
Model 1	1.00 (Ref)	0.97	(0.67,1.42)	0.90	0.95	(0.65,1.39)	0.80	0.80
Model 2	1.00 (Ref)	0.97	(0.66,1.44)	0.91	0.95	(0.64,1.40)	0.81	0.96
Model 3	1.00 (Ref)	0.97	(0.66,1.43)	0.89	0.95	(0.63,1.43)	0.82	0.96

Model 1: unadjusted, Model2: Age, sex, education (categorical), Marriage, life-style, smoking; Model 3: Model 2 + physical activity, total energy intake.

Table 5 shows the unadjusted and adjusted odds ratios for central obesity across the tertiles of dietary pattern scores according to WC (over 102cm for men and over 88cm for women) and waist to hip ratio (above 0.90 for men and above 0.85 for women) definitions. The results of the analysis based on central obesity showed that there was no significant relationship between adherence to prudent dietary pattern and increasing the waist to hip ratio (OR: 0.92, 95%CI = 0.64, 1.32, *P* value = 0.68). And, no significant relationship was observed between adherence to potatoes and eggs pattern and increasing the waist to hip ratio (OR: 0.78, 95%CI = 0.55, 1.11, *P* value = 0.17). In addition to, we found no significant relationship between Western dietary pattern and the chance of increasing the waist to hip ratio (OR: 0.93, 95%CI = 0.64, 1.34, *P* value = 0.69). Finally, there was no significant relationship with WC in any dietary pattern, in the unadjusted models, which remained insignificant after controlling for confounding factors.

**Table 5:** Odds ratio (OR)s and 95% confidence intervals(CI) for central obesity across tertiles (T) of dietary patterns score

	**Tertiles of dietary pattern score**							
	**T1**	**T2**		***P*** ** value**	**T3**		***P*** ** value**	**P trend**
		**OR**	**95% CI**		**OR**	**95% CI**		
**Participants**	**(n=266)**	**(n=283)**		**(n=284)**		
Waist-circumference (cm) above 102 cm for men and above 88 cm for women								
Prudent								
Model 1	1.00 (Ref)	0.99	(0.70,1.39)	0.96	1.007	(0.71,1.41)	0.96	0.96
Model 2	1.00 (Ref)	1.02	(0.72,1.44)	0.90	1.007	(0.71,1.42)	0.96	0.99
Model 3	1.00 (Ref)	0.98	(0.69,1.39)	0.92	0.95	(0.66,1.36)	0.80	0.96
Potatoes and eggs								
Model 1	1.00 (Ref)	0.87	(0.62,1.23)	0.45	0.83	(0.59,1.17)	0.30	0.30
Model 2	1.00 (Ref)	0.84	(0.59,1.19)	0.33	0.82	(0.58,1.17)	0.28	0.50
Model 3	1.00 (Ref)	0.85	(0.60,1.20)	0.35	0.79	(0.55,1.13)	0.19	0.42
Western								
Model 1	1.00 (Ref)	1.04	(0.74,1.48)	0.78	1.37	(0.97,1.94)	0.06	0.06
Model 2	1.00 (Ref)	1.03	(0.73,1.47)	0.84	1.36	(0.96,1.93)	0.08	0.16
Model 3	1.00 (Ref)	1.02	(0.71,1.45)	0.91	1.30	(0.90,1.88)	0.16	0.29
Waist to hip ratio above 0.90 for males and above 0.85 for females								
Prudent								
Model 1	1.00 (Ref)	0.80	(0.56,1.13)	0.20	0.97	(0.69,1.38)	0.90	0.90
Model 2	1.00 (Ref)	0.83	(0.58,1.17)	0.30	0.99	(0.70,1.40)	0.96	0.50
Model 3	1.00 (Ref)	0.81	(0.57,1.15)	0.23	0.92	(0.64,1.32)	0.68	0.49
Potatoes and eggs								
Model 1	1.00 (Ref)	0.78	(0.55,1.10)	0.16	0.81	(0.57,1.14)	0.23	0.23
Model 2	1.00 (Ref)	0.76	(0.53,1.08)	0.12	0.80	(0.56,1.14)	0.22	0.27
Model 3	1.00 (Ref)	0.77	(0.54,1.09)	0.14	0.78	(0.55,1.11)	0.17	0.26
Western								
Model 1	1.00 (Ref)	1.04	(0.74,1.47)	0.79	1.03	(0.73,1.45)	0.86	0.86
Model 2	1.00 (Ref)	1.03	(0.72,1.45)	0.86	1.002	(0.70,1.41)	0.99	0.98
Model 3	1.00 (Ref)	1.007	(0.71,1.42)	0.96	0.93	(0.64,1.34)	0.69	0.89

Model 1: unadjusted, Model2: Age, sex, education (categorical), Marriage, life-style, smoking; Model 3: Model 2 + physical activity and total energy intake.

## Discussion

We identified three main dietary patterns by factor analysis: “Prudent”, “egg and potato”, and “Western”. The results of this study showed that there was no significant relationship between any of the major dietary patterns in the unadjusted models with general and central obesity, and this lack of significant association remained unchanged following adjustment for age, sex, smoking status, education, marital status, life status, physical activity, and total energy intake in the logistic regression model.

Considering meal levels in dietary pattern analysis has many benefits relative to analyzing nutrients or foods alone. Such an approach facilitates the understanding of the synergistic effects of nutrients and foods, which enables easy to recommend findings for community health.^31^ In addition, given that the pattern of obesity is different according to the type of diet in people, and the dietary intake of the individuals varies at the meal level, therefore, examining this relationship at the meal levels is necessary to simplify meal recommendations and to help preventing obesity. In this study, the three major dietary patterns identified at the dinner meal were as follows: dietary pattern 1 “prudent”, which was rich in bread and cereals, salt and fat and also included low use of fruits and vegetables, poultry, and fish, and nuts and legumes, dietary pattern 2 “potatoes and eggs”, which included high consumption of potatoes and eggs, but low consumption of dairy, nuts and legumes, and dietary pattern 3 “ Western “, which was associated had high intake of red or processed meats, sauces, soft drink, and bread and grains and low consumption of poultry and fish. These three dietary patterns, together, accounted for 38.74 percent of the total distribution of variance in the study population.

A study in Japan examined the dietary pattern of 15,618 adult participants in breakfast, lunch, and dinner meals. The “prudent” dietary pattern in our study was similar to the third pattern of Japanese people (other grains/fat).^32^ Moreover, there was similarities between dietary patterns and those identified by another study in Germany, where Schwedhelm et al. identified four dietary patterns, including prudent, Western, traditional, and cereals and legumes. The “prudent” dietary pattern in our study were relatively similar to the traditional pattern (high intake of bread, processed meat, butter, sugar, confectionery, cakes and cookies and low intake of water) and the “Western” dietary pattern (high intake of potatoes, cabbage, red meat, beer, sauces and condiments and low intake of fresh fruits, milk and dairy products and tea) in the German population.^33^ Santos et al. also investigated dietary patterns in association with health outcomes at meal levels, and reported three dietary patterns at breakfast, five dietary patterns at lunch, and four patterns at dinner. However, there was a very little similarity among the dietary patterns of the present study to patterns observed in Brazilian society, which may be attributed to the differences in the culture, tradition, and eating habits of the Iranian people compared to Brazilian people.^34^ In addition, in some studies, dietary patterns were measured based on a FFQ; although the FFQ is considered an appropriate tool for collecting dietary data in large epidemiological studies, it has both systematic and random error that may affect estimates of diet-disease associations.

In the present study, no association was found between the “prudent” dietary pattern and obesity. The lack of association may be attributed to the presence of different food items in this pattern. The protective effect of a healthy dietary pattern may be due to the effect of high fiber and complex carbohydrates, low glycemic index and low energy dense foods, such as vegetables, fruits and nuts.^35,36^ There is also sufficient evidence highlighting that fruits and vegetables contain a number of biologically active components, including vitamins, sterols, phenolic compounds, and fiber, which may independently protect against various diseases.^37^ In addition, some studies have shown that extreme consumption of fruits and vegetables can help to reduce the risk of weight gain in individuals .^38^ On the other hand, consuming higher amounts of fat, as constituent in unhealthy dietary patterns, is a risk factor for weight gain.^39^ However, different food groups in the “prudent” pattern may have weakened the potential relationship to the risk of obesity or overweight in the present study.

We found that there was no significant relationship between the “Western” dietary pattern and obesity. However, a positive association between the “Western” dietary pattern with overweight and obesity is supported by many previous studies ^40,41^; although, consistence with our findings, it has been reported that no association exists between “Western” dietary patterns and BMI.^42,43^ Meal compounds of such food pattern (Western -style) have been associated with metabolic abnormalities due to the relatively high levels of fat, sodium and sugar ^44^, which are well associated with excess weight gain. Therefore, the lack of a positive association in this study was unexpected. In general, it is difficult to compare previous findings with the existing results, particularly due to the characteristics of diet patterns. In addition, confounding factors may also play a mediating role.

In the present study, no significant relationship was found between the dietary pattern “potato and the egg” and obesity, which is similar to the healthy dietary pattern. Indeed, the healthy dietary pattern which contains high amounts of fiber, low energy dense foods, and high amounts of water, may play an important role in reducing the prevalence of obesity.^45^ Some previous studies have reported similar findings.^46,47^ However, most studies have reported an inverse relationship between a healthy dietary pattern and the risk of obesity.^11,48^ As noted earlier, because the identified dietary patterns are a combination of food groups that may be distributed differently in each study they cannot be exactly compared in terms of nutrient intake. In a number of studies, people who did not consume dinner were more likely to be obese.^49^ On the other hand, some evidence exists to suggest a positive relationship between eating at night and obesity, especially when dinner is eaten late.^50^ An explanation for this relationship might be attributed to the accumulation of energy, in the form of glycogen, after eating a carbohydrate-rich dinner late at night, suggesting that it might prevent the rapid use of this energy, and thus support its accumulation.^51^

One of the strengths of the present study is the use of validated tools to collect data; in addition to the recruitment of a large sample size compared to other studies which have investigated dietary pattern in Iran. Furthermore, all areas of Tehran were included in this study, permitting the inclusion of various socio-economic, education, and welfare levels, and other variables affecting the outcome.

Although the present study provides a novel contribution to the literature, there are some limitations worth considering. For instance, this study was cross-sectional by design, preventing causal inferences to be made. In addition, recall bias may be occur due to data collection using the questionnaire; whilst the accuracy of this information also depends on the person’s memory and level of education. Furthermore, one of the limitations of analyzing dietary patterns is its’ subjective classification; although such classifications were created using collaborative expertise and prior evidence. Finally, there may be significant differences in dietary patterns according to geographic area, race, and culture in different populations. Then, according to all of these limitations contributed to identifying dietary patterns and recording dietary data, and possible differences in characteristics of adults living in Tehran from other parts of Iran, generalizability of the dietary patterns and its relation to obesity to the whole population of Iranian adults is not possible.

## Conclusion

The results of this study do not support a possible relationship between major dietary patterns at dinner with general and central obesity. However, the presented findings should be confirmed in prospective studies.

## Acknowledgments

Authors thanks all those who participated in this study.

## Competing interest

None.

## Ethical approval

This study was conducted according to the guidelines laid down in the Declaration of Helsinki and all procedures involving research study participants were approved by the ethics committee of Tehran University of Medical Sciences (IR.TUMS.VCR.REC.1397.157). Written informed consent was obtained from all subjects/patients..

## Funding

This maunuscript has been granted by Tehran University of Medical Sciences (Grant No: 40186).
